# AMP-Activated Kinase (AMPK) Activation by AICAR in Human White Adipocytes Derived from Pericardial White Adipose Tissue Stem Cells Induces a Partial Beige-Like Phenotype

**DOI:** 10.1371/journal.pone.0157644

**Published:** 2016-06-20

**Authors:** Omar Abdul-Rahman, Endre Kristóf, Quang-Minh Doan-Xuan, András Vida, Lilla Nagy, Ambrus Horváth, József Simon, Tamás Maros, István Szentkirályi, Lehel Palotás, Tamás Debreceni, Péter Csizmadia, Tamás Szerafin, Tamás Fodor, Magdolna Szántó, Attila Tóth, Borbála Kiss, Zsolt Bacsó, Péter Bai

**Affiliations:** 1 Department of Medical Chemistry, Faculty of Medicine, University of Debrecen, Debrecen, H-4032, Hungary; 2 Department of Biochemistry and Molecular Biology, Faculty of Medicine, University of Debrecen, Debrecen, H-4032, Hungary; 3 Department of Biophysics and Cell Biology, Faculty of Medicine, University of Debrecen, Debrecen, H-4032, Hungary; 4 Department of Cardiology, Faculty of Medicine, University of Debrecen, Debrecen, H-4032, Hungary; 5 Department of Dermatology, Faculty of Medicine, University of Debrecen, Debrecen, H-4032, Hungary; 6 MTA-DE Cell Biology and Signaling Research Group, Debrecen, H-4032, Hungary; 7 MTA-DE Lendület Laboratory of Cellular Metabolism, Debrecen, H-4032, Hungary; 8 Research Center for Molecular Medicine, University of Debrecen, Debrecen, H-4032, Hungary; Hungarian Academy of Sciences, HUNGARY

## Abstract

Beige adipocytes are special cells situated in the white adipose tissue. Beige adipocytes, lacking thermogenic cues, morphologically look quite similar to regular white adipocytes, but with a markedly different response to adrenalin. White adipocytes respond to adrenergic stimuli by enhancing lipolysis, while in beige adipocytes adrenalin induces mitochondrial biogenesis too. A key step in the differentiation and function of beige adipocytes is the deacetylation of peroxisome proliferator-activated receptor (PPARγ) by SIRT1 and the consequent mitochondrial biogenesis. AMP-activated protein kinase (AMPK) is an upstream activator of SIRT1, therefore we set out to investigate the role of AMPK in beige adipocyte differentiation using human adipose-derived mesenchymal stem cells (hADMSCs) from pericardial adipose tissue. hADMSCs were differentiated to white and beige adipocytes and the differentiation medium of the white adipocytes was supplemented with 100 μM [(2R,3S,4R,5R)-5-(4-Carbamoyl-5-aminoimidazol-1-yl)-3,4-dihydroxyoxolan-2-yl]methyl dihydrogen phosphate (AICAR), a known activator of AMPK. The activation of AMPK with AICAR led to the appearance of beige-like morphological properties in differentiated white adipocytes. Namely, smaller lipid droplets appeared in AICAR-treated white adipocytes in a similar fashion as in beige cells. Moreover, in AICAR-treated white adipocytes the mitochondrial network was more fused than in white adipocytes; a fused mitochondrial system was characteristic to beige adipocytes. Despite the morphological similarities between AICAR-treated white adipocytes and beige cells, functionally AICAR-treated white adipocytes were similar to white adipocytes. We were unable to detect increases in basal or cAMP-induced oxygen consumption rate (a marker of mitochondrial biogenesis) when comparing control and AICAR-treated white adipocytes. Similarly, markers of beige adipocytes such as TBX1, UCP1, CIDEA, PRDM16 and TMEM26 remained the same when comparing control and AICAR-treated white adipocytes. Our data point out that in human pericardial hADMSCs the role of AMPK activation in controlling beige differentiation is restricted to morphological features, but not to actual metabolic changes.

## Introduction

The energy balance of an organism depends on the net of energy intake and energy expenditure. The disequilibrium between energy uptake and energy expenditure has causative role in the pathogenesis of metabolic diseases [1–5]. Energy expenditure stems from the energy deliberated by biochemical processes, physical activity or the action of mitochondria-rich tissues such as skeletal muscle, brown adipose tissue or cardiac muscle [6]. Recently a novel cell type called beige, or brite (blended from *br*own and wh*ite*) adipocytes were identified in white adipose tissue (WAT) that seems to play an indispensable role in energy expenditure [7, 8].

Inactive beige cells morphologically appear as normal WAT cells, however upon adrenergic stimulation beige adipocytes do not only enhance lipolysis but upregulate mitochondrial biogenesis and mitochondrial oxidation and futile cycle of creatine phosphate generation at the same time [7, 9–11]. It is conceivable therefore that due to the proportions of WAT in the human body, beige adipocytes may have similar importance in energy expenditure comparable to skeletal muscle. Importantly, functional beige adipocytes were shown in humans too [7, 12], moreover these cells are transplantable [13].

The induction of beige adipocyte had been demonstrated in humans upon cold exposure [7] suggesting that beige cells are intricately embedded into the neuroendocrine regulation of metabolism. Several hormones were shown to regulate the metabolism and differentiation of beige adipocytes (e.g. Irisin, FGF21, NRG4, BMP4, GLP-1) [7, 14–18] together with signals from AgRP neurons and the serotoninergic system [19, 20]. Importantly, thiazolidinediones or fibrates were shown to induce browning too [21, 22]. Surprisingly, the immune system is also implicated in beige adipocyte differentiation, the tolerogenic, anti-diabetic type 2 macrophage polarization favors beige differentiation [23].

Activating stimuli in beige adipocytes induce SIRT1 that deacetylates and hence activates PPARγ declutching a set of mitotropic events involving peroxisome proliferator activated receptor cofactor-1α (PGC-1α) that lead to the enhanced mitochondrial biogenesis and oxidation [21] that is fine-tuned by a set of micro-RNAs [15, 24]. AMP-activated protein kinase (AMPK) is an upstream activator of SIRT1 [25]. This protein kinase is activated by the shortage of cellular energy stores and upon activation AMPK initiates cellular programs that silence anabolic processes to save energy and induce catabolism to resolve the energy crisis [25]. Importantly, AMPK activation had been implicated in brown adipocyte differentiation and function [26]. The known involvement in brown adipocyte function and mitochondrial biogenesis made it very likely that AMPK could be involved in the function of beige adipocytes too. In the present study we set out to assess that possibility.

## Methods

### Chemicals

Unless otherwise stated, all chemicals were from Sigma-Aldrich (St. Louis, MO, USA).

### Ethical statement

Human adipose-derived mesenchymal stem cells (hADMSCs) were isolated from pericardial adipose tissue of patients who underwent a planned heart surgery (e.g. valve surgery, coronary bypass surgery, Batista operation). No exclusions were applied regarding BMI, age, gender or medications of the patients. Written informed consent from all participants was obtained before the surgical procedure. The study protocol was approved by the Ethics Committee of the University of Debrecen (Hungary) and the Medical Research Council Committee of Human Reproduction (No. 3992-2013/DEOEC RKEB/IKEB). All experiments were carried out in accordance with the approved ethical guidelines and regulations.

### Isolation, culture and differentiation of human adipose derived mesenchymal stem cells (hADMSCs)

On the day of heart surgery the pericardial adipose tissue specimen were processed as described in [14]. Samples were digested in PBS with 120U/ml collagenase for 1 hour at 37°C and filtered through a sieve with pore size 100μm. Isolated hADMSCs were resuspended in DMEM-F12 medium containing 10% FBS (Gibco) and seeded to the appropriate vessels. After cell culture reached confluency, differentiation was initiated. White adipogenic differentiation was carried out using the protocol of Fischer-Posovszky and co-workers [27]. For brown adipogenic differentiation cells were treated according to Elabd and co-workers [28]. For differentiation FBS-free medium was used. To assess the result of AMPK activation, white adipocytes were differentiated in the presence of 100 μM [(2R,3S,4R,5R)-5-(4-Carbamoyl-5-aminoimidazol-1-yl)-3,4-dihydroxyoxolan-2-yl]methyl dihydrogen phosphate (AICAR) (Tocris, Bristol, UK) [29]. Cells were assayed after 14 days differentiation.

### Image acquisition, recognition and texture analysis

Cellular texture analysis was performed using laser scanning cytometry on differentiated cells as described in [14, 30].

hADMSCs were plated on Ibidi eight-well μ-slides and differentiated as previously described. On the day of measurement, cells were stained with Hoechst 33342 (50 μg/ml) and with Nile Red (NR, 25 μg/ml) for 20 minutes. Cells were washed once with phosphate buffered saline (PBS) and then kept in fresh medium.

Images were obtained by using iCys (CompuCyte) laser scanning cytometer (Thorlabs Imaging Systems, Sterling, VA, USA) according to the protocol of Doan-Xuan and co-workers [30]. Images were processed and analyzed by CellProfiler (The Broad Institute of MIT, MA). Hoechst-stained nuclei from both differentiated and undifferentiated cells were first identified and marked as primary objects. Differentiated adipocytes were later recognized by their lipid droplet specific Nile Red absorption signal. Cells above a preset threshold were considered as differentiated adipocytes. The threshold was calculated from the efficiency of NR staining in every measurement where intensity values were plotted against individual cell counts. Staining efficiency of undifferentiated preadipocytes were used as a baseline. Texture analysis per identified objects was done with built-in modules in CellProfiler. Parameter entropy measured the randomness of intensity distribution; sum entropy informed about the number of lipid droplets. Parameter variance measured the difference between intensity of the central pixel and its neighborhood; sum variance depicted the size of lipid droplets.

### Analysis of mitochondrial fusion

The structure of the mitochondrial network in cells changes its shape as a function of cellular bioenergetics (e.g. fasting or feeding) and environmental stimuli [31, 32]. The structure of the mitochondrial network changes between a fully fused (long, interconnected mitochondrial tubes) and fully fragmented state (smaller, individual mitochondria with dotted appearance) as hypothetical endpoints, where the fused state is associated with better mitochondrial oxidative activity [31, 32].

Preadipocytes were seeded on Ibidi eight-well μ-slides and differentiated as previously described. On the day of analysis cells were stained with Mitotracker Red (Thermo Scientific, MA, USA) using a working concentration of 100 nM for 20 minutes at 37°C. That dye charges the mitochondria enabling the visualization of the mitochondrial network. Cells were washed once with PBS and then kept in fresh medium. Images were taken by a Leica TCS SP8 confocal microscope (Leica Microsystems, Wtzlar, Germany).

**Fig 1:**
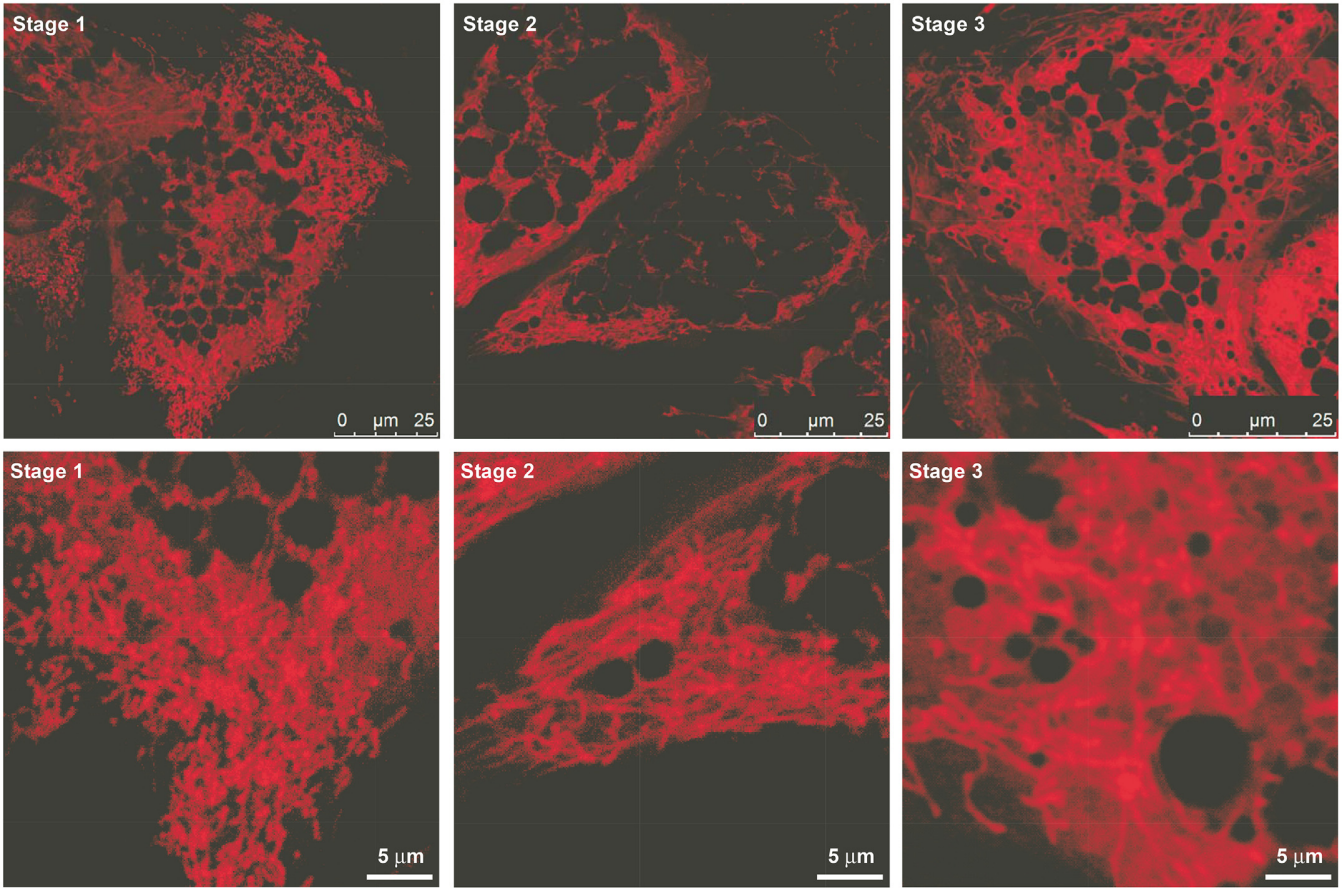
Representation of the stages of mitochondrial fusion in differentiated hADMSCs. The bottom line is an enlarged part of the upper image. Scale bars are on the images.

Differentiated cells were grouped into three categories according to the morphology of their mitochondrial network. When characterizing a sample, we analyzed and scored 100 cells and each cell was scored between 1 to 3 as a function of mitochondrial network morphology. Cells with dotted staining were scored 1 (Stage 1 on the figures) implying no or minimal fusion between mitochondria. In cells scored 2 (Stage 2 on the figures) the mitochondrion-specific staining draws a much more elongated mitochondria as a result of elevated fusion. Cells scored 3 (Stage 3 on the figures) have a network-like structure formed by the fusion of mitochondria as a consequence of high fusion activity. Examples for the different extent of fusion are shown on Fig 1.

### Western blotting

Protein extraction, SDS-PAGE and Western blotting were performed as in [33]. Membranes were probed with polyclonal phospho-acetyl-CoA-carboxylase antibody (pACC, 1:500) (Cell Signaling, MA, USA), ACC (*Cell signaling*, anti-rabbit monoclonal antibody, 1:1000), phospho-AMPKα (Thr172, *Cell signaling*, anti rabbit polyclonal, 1:1000) and AMPKα (*Sigma Aldrich*, anti-rabbit polyclonal antibody, 1:1000) overnight at 4°C as a downstream sign of AMPK activity and monoclonal Anti-β-Actin−Peroxidase antibody (1:20000) for 1h at room temperature. For pACC antibody the secondary antibody was IgG peroxidase HRP conjugate. Immunoreactions were detected by enhanced chemiluminescence (West Pico ECL Kit, Thermo Scientific).

### Determination of cellular oxygen consumption

Oxygen consumption was measured using an XF96 oximeter (Seahorse Biosciences, North Billerica, MA, USA). Cells were seeded and differentiated in 96-well XF96 assay plates. On the day of measurement, after recording the baseline oxygen consumption, cells received a single bolus dose of dibutyril-cAMP (500 μM final concentration) simulating adrenergic stimulation. Then, stimulated oxygen consumption was recorded every 30 minutes. The final reading took place at 7 h post-treatment. As a last step, cells received a single bolus dose of antimycin A (10 μM) for baseline correction [14, 34].

### Reverse transcription-coupled quantitative PCR (RT-qPCR)

Total RNA preparation, reverse transcription, and RT-qPCR were performed as in [35]. Total cellular RNA was isolated using TRIzol Reagent (Molecular Research Center, OH, USA). Primers are summarized in Table 1. All reactions were run in a LightCycler 480 (Roche Diagnostics, Mannheim, Germany) instrument using SYBR green chemistry except for TBX1 where primers and probes were designed and supplied by Applied Biosystems (Taqman Hs00271949_m1, Applied Biosystems). Expression was normalized to the geometric mean of two control genes (G6PD- glucose-6-phosphate dehydrogenase, 36B4- ribosomal protein, large, P0). Gene expression values were calculated based on the ΔΔCt method, where white samples were designated as calibrator.

**Table 1:** Sequence of DNA primers used for gene expression analysis.

Gene	Forward primer	Reverse primer
UCP1	AACGAAGGACCAACGGCTTTC	GGCACAGTCCATAGTCTGCCTTG
CIDEA	TCTCCAACCATGACAGGAGCAG	AATGCGTGTTGTCTCCCAAGGT
PRDM16	CACTGTGCAGGCAGGCTAAGAA	AGAGGTGGTTGATGGGGTGAAA
TMEM26	ACCTCCCATGTGTGGACATCCT	ACCAACAGCACCAACAACCTCA
G6PD	GCCTCATCCTGGACGTCTTCT	GGTGCCCTCATACTGGAAACC
36B4	CCATTGAAATCCTGAGTGATGTG	GTCGAACACCTGCTGGATGAC

### Statistical analysis

The appropriate test and the details of cohort are given in the figure legends.

## Results

### AICAR-induced AMPK activation leads to beige-like morphological changes in hADMSCs-derived white adipocytes

**Fig 2:**
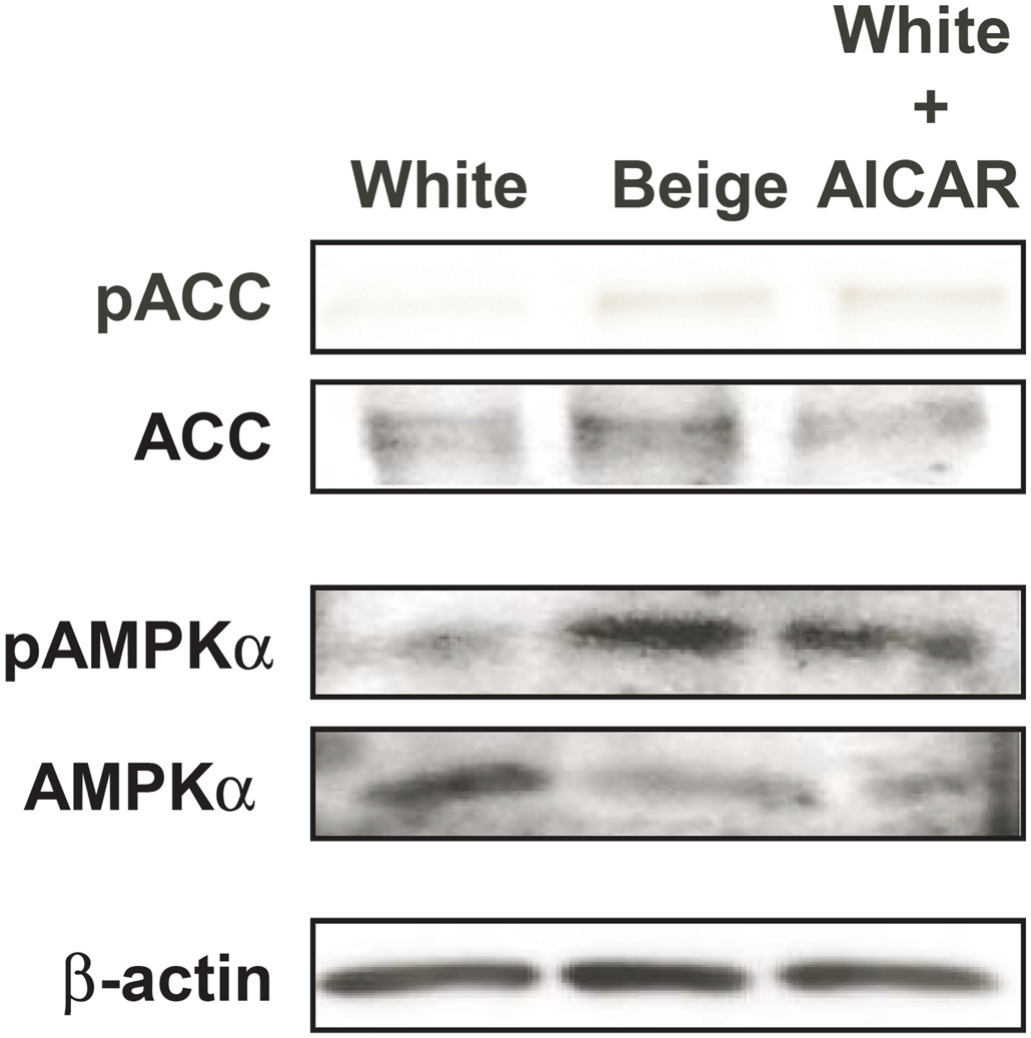
Evaluation of AMPK activity in differentiated adipocytes. AMPK activity was evaluated in white, beige and white adipocytes treated with 100 μM AICAR by assessing ACC phosphorylation (pACC), total ACC, phosphor-AMPKα and total AMPKα levels by Western blotting.

Each hADMSCs cell line (= each individual) was differentiated in three directions, namely towards beige adipocytes, white adipocytes and AICAR-treated white adipocytes. First we checked AMPK activity in the three groups at the end of the differentiation. AMPK activity was higher in beige than in white adipocytes (Fig 2). Importantly, the treatment of white adipocytes with 100 μM AICAR enhanced AMPK activity almost to the same extent as in beige adipocytes (Fig 2) that suggested a role for AMPK in beige adipocyte differentiation and function.

**Fig 3:**
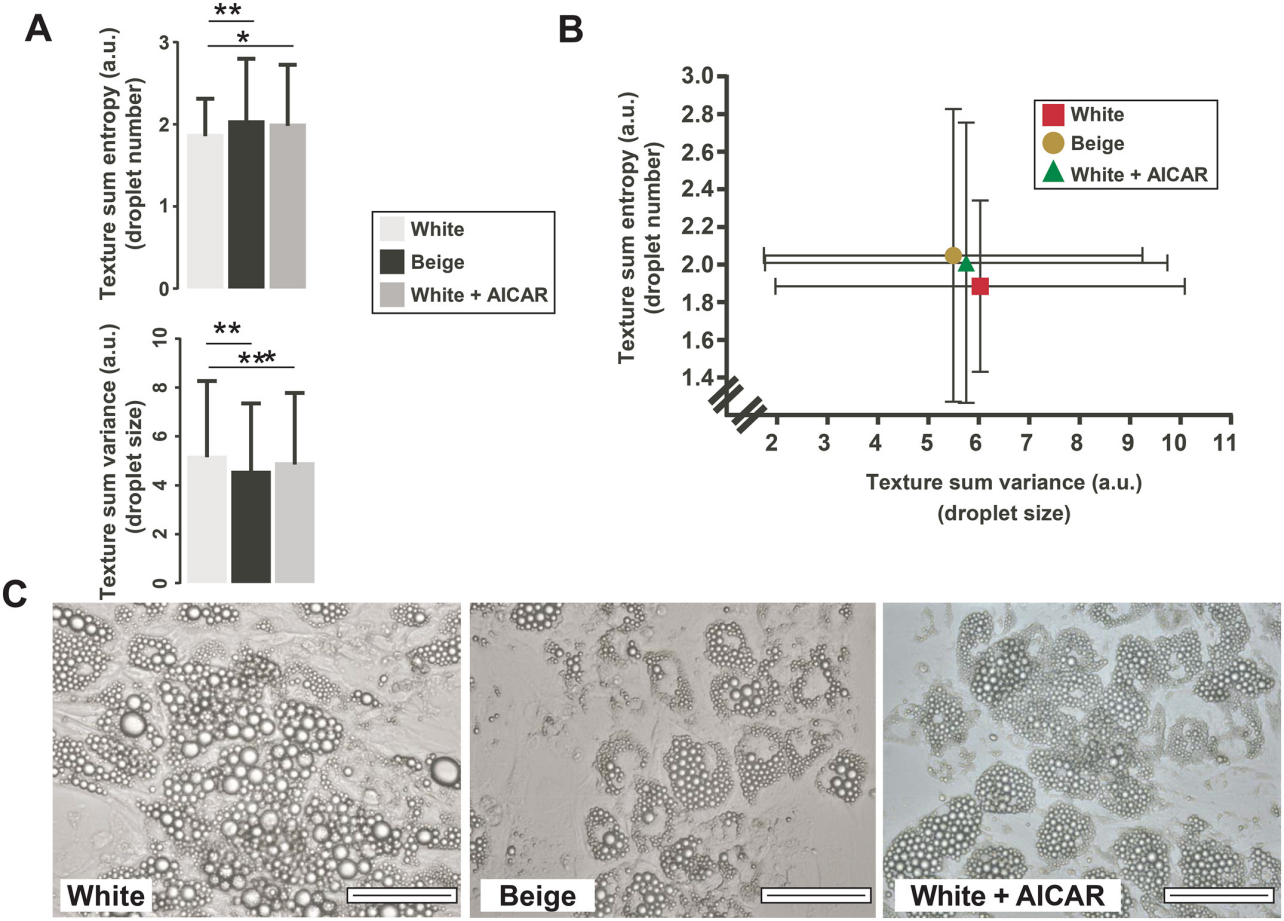
Morphology changes in white adipocytes upon AICAR treatment. Lipid droplet morphology was evaluated by laser scanning microscopy (LSC). **(A)** The sum entropy of the texture and sum variance of the texture was assessed (median ± quartiles, n = 3); statistical significance was assessed using the Kruskal-Wallis test. *, ** and *** indicate statistically significant difference between the indicated groups at p<0.05, p<0.01 and p<0.001, respectively. **(B)** The sum entropy of the texture and sum variance of the texture are plotted against each other (mean ± SD, n = 3). **(C)** Representative images of one donor are presented. The scale bar represents 100 μm.

Next we evaluated morphological changes upon AICAR treatment. Previous studies have shown that beige adipocytes have smaller lipid droplets in larger numbers as white adipocytes [14, 30]. Laser scanning cytometry (LSC) was proved to be an efficient tool to characterize adipocyte morphology [14, 30], therefore we performed LSC analysis on our samples. We have reproduced the previously-described size and number differences of the lipid droplets between white and beige adipocytes [14, 30] (Fig 3A–3C). The treatment of white adipocytes with AICAR reduced the average size of lipid droplets and concomitantly increased the total number of droplets (Fig 3A–3C).

**Fig 4:**
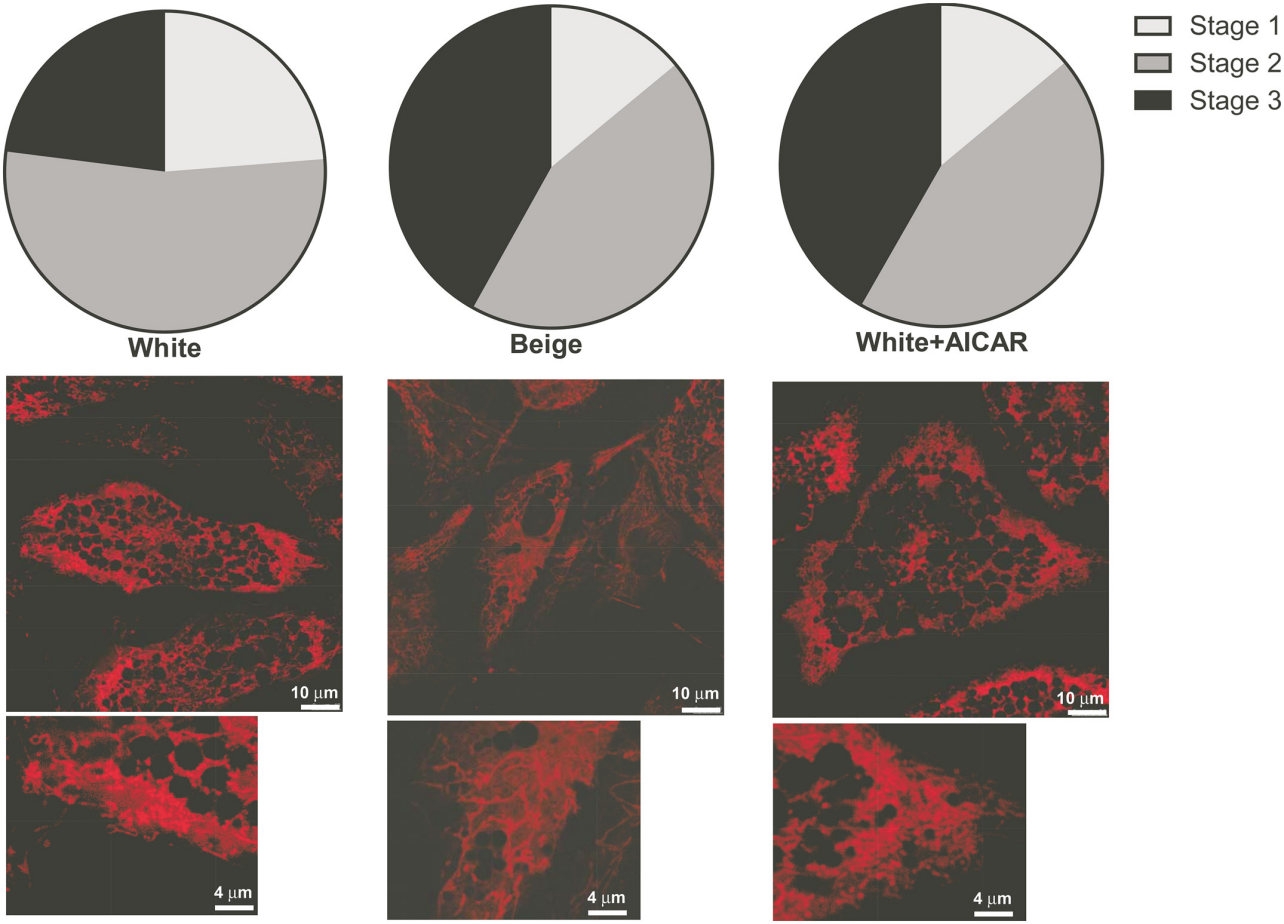
Assessment of mitochondrial network in differentiated adipocytes. Mitochondria in differentiated adipocytes were charged by Mitotracker Red and mitochondrial network was assessed by confocal microscopy; cells were scored as described in the Materials section. The proportions of the fragmented and fused mitochondria were plotted as pie charts (n = 4). To assess statistical significance chi square test was performed, where the distribution of the white adipocytes was the expected distribution. The distribution of the AICAR-treated white adipocytes and beige adipocytes were significantly different from the untreated white adipocytes (p<0.01), while there was no statistical difference between AICAR-treated white adipocytes and beige adipocytes. Representative images of the mitochondrial network is presented on the figure. A part of these images had been enlarged.

The biological function of beige adipocytes depend on mitochondrial biogenesis and the upregulation of mitochondrial oxidation, therefore we continued our experiments by assessing mitochondrial function. First we assessed mitochondrial morphology that changes in accordance with the mitochondrial oxidative function [36–39]. The mitochondrial network was assessed by fluorescent microscopy. The mitochondrial network was more fused when cells were differentiated towards beige adipocytes as compared to white adipocytes (Fig 4). Upon the activation of AMPK by AICAR, the mitochondrial network of adipocytes became more similar to the one of beige adipocytes that was dominated by fused mitochondria suggesting enhanced mitochondrial activity (Fig 4).

### AICAR treatment of hADMSCs-derived white adipocytes does not yield functional beige adipocytes

**Fig 5:**
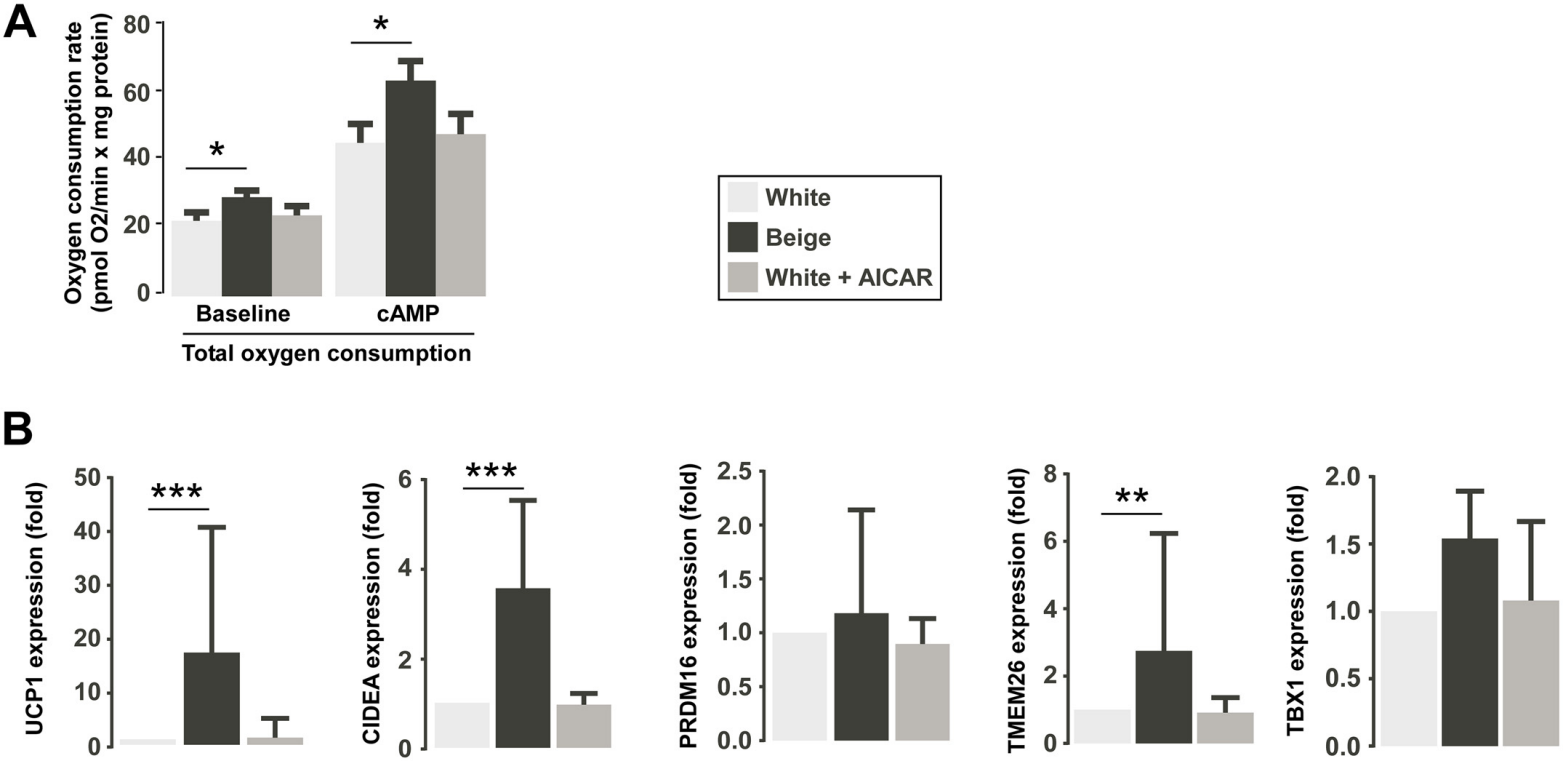
Assessment of mitochondrial function in differentiated adipocytes. **(A)** Mitochondrial oxygen consumption was determined using the Seahorse oximeter. One representative donor is shown (mean ± SD), statistical significance was determined using the one-way ANOVA. * indicate statistically significant difference between the indicated groups at p<0.05. **(B)** The expression of the indicated genes were determined in RT-qPCR reactions (n = 8 except for TBX-1, where n = 3, median and quartiles are plotted). Statistical significance was determined using one-way ANOVA. ** and *** indicate statistically significant difference between the indicated groups at p<0.01 and p<0.001, respectively. Abbreviations are in the text.

The experiments in the previous chapter suggest enhanced mitochondrial oxidation, therefore we measured mitochondrial oxygen consumption. In accordance with our previous report [14], beige adipocytes displayed higher basal and cAMP-stimulated oxygen consumption rate as white adipocytes, however AICAR-treatment of white adipocytes did not increase oxygen consumption (Fig 5A).

This surprising finding prompted us to assess validated markers of beige differentiation [40]. We measured the mRNA levels of uncoupling protein-1 (UCP1) that is a mitochondrial internal membrane protein that bypasses ATP synthase and creates heat out of the proton gradient, CIDEA, PRDM16 that are transcriptional co-activators, T-box protein 1 (TBX-1) that is a transcription factor and TMEM26, a transmembrane protein of unknown function. Out of these markers TBX-1 is known as a validated beige marker, the rest appear both in beige and brown adipocytes [40–42]. All markers were expressed at higher levels in beige cells as compared to white adipocytes, however we were unable to detect increases upon AICAR treatment as compared to white adipocytes (UCP1 was slightly induced) (Fig 5B).

Taken together, these data suggest that white adipocytes that are differentiated from pericardium-derived hADMSCs, upon treatment AICAR do not become functional beige cells. They display only certain morphological features, but apparently in terms of biochemical functions, AICAR-treated cells rather behave as white adipocytes.

## Conclusions

Beige adipocytes represent a newly described pool of adipocytes with physiologically important capacity of oxidation that is implicated in the pathology of type II diabetes and probably a myriad of other metabolic diseases [7, 43]. Qiang and colleagues have identified the SIRT1 –PPARγ pathway as the central regulator of beige adipocyte differentiation and function [21]. SIRT1, a protein deacetylase, is the member of the sirtuin protein family that was implicated in several human metabolic and degenerative diseases [2, 44]. SIRT1 has central role in the adaptation to fasting and other nutritional or environmental stress. SIRT1 achieves its effects through deacetylating key transcription factors (PGC-1α, FOXO1, FOXO3, p53) that then initiate transcription programs culminating in the suppression of cellular synthetic processes and the induction of cellular catabolism and mitochondrial biogenesis [2, 44]. SIRT1 works in collaboration with other cellular energy sensors such as mechanistic Target of Rapamycin (mTOR), hypoxia-inducible factors (HIFs), nuclear respiratory factors (NRFs) or AMPK [2, 44–47]. AMPK has very similar role to SIRT1, when cellular energy charge decreases marked by increase in AMP levels and the decrease of ATP, AMPK is activated and shuts down energy-consuming anabolic processes and turns on mitochondrial oxidation and catabolism to replenish cellular energy [25, 48].

SIRT1 and AMPK not only display functional synergy, but their activity is cross-regulated [25, 49–53]. Several investigators have shown that AMPK and SIRT1 act on the same transcription factors (e.g. PGC-1α) that requires both AMPK-mediated phosphorylation and SIRT1-mediated deacetylation for activation and the subsequent induction of mitochondrial biogenesis [25]. Furthermore, AMPK and SIRT1 are also cross-connected through modulating NAMPT and hence cellular NAD^+^ salvage [52]. Since the same target transcription factors and SIRT1 were implicated in the differentiation and function of beige cells (e.g. PGC-1α) [15, 17, 21, 22, 54, 55] it was logical to assess the function of AMPK, which is situated upstream of SIRT1, in beige differentiation. This hypothesis was further supported by the fact that AMPK activation is important in brown adipose tissue differentiation, moreover induces differentiation of white adipocyte precursors to brown adipocytes [55–61].

We used AICAR for the activation of AMPK [29] and found typical morphological features of beige cells are formed upon AICAR treatment. However, the very necessary induction of mitochondrial biogenesis and the increased expression of the markers of beige differentiation (TBX-1, UCP1, TMEM26, PRDM16, CIDEA) did not take place. It seems that AMPK activation does not induce the formation of functional beige cells in pericardial hADMSCs, but AMPK activation is restricted to the induction of morphological changes. The actual cause of the restricted capability of AMPK in inducing beige differentiation remains elusive. Terminal differentiation of beige cells depend on SIRT1 activation [21], while AMPK is capable of activating SIRT1 [25]. The interconnection between AMPK and SIRT1 relies on the modulation of small molecule enzyme cofactors such as NAD^+^ [25, 48]. Characteristic differences in the metabolism of these cofactors or low AMPK expression in beige cells may explain blunted response to AMPK activation, although to verify these hypothesis require further investigations.

It is a question whether the restricted role of AMPK is true for adipose tissue-derived stem cells of any origin or is it specific for only (a) certain fat depot(s). Indeed, the expression of PRDM16 and TBX-1 in beige cells versus white cells is lower in our study than in another study using stem cells from subcutaneous adipose tissue [14]. Yet the question cannot be answered with certainty, however the known differences between adipose tissue depots [62, 63] makes it likely that AMPK activation may have a more pronounced effect in other adipose tissue depots. It should be noted, however, that this study is the first to report that pericardial adipose tissue-derived stem cells can be differentiated to beige adipocytes.

Although several known external stimuli (hormones [7, 14–18], neuronal stimuli [19, 20] or drugs [21, 22]) may activate beige cells, the intracellular signal transduction pathways that translate these signals are still to be elucidated. It seems that β-adrenergic stimulus and signals induce beige adipocyte function [64, 65]. However, serotonin (5HT) had been shown to blunt thermogenic activity in brown and beige cells in mice [66, 67]. Our data nominate the energy and nutrient sensing system as possible actors in beige function and in signal transduction. Along the same line, there is a vast area of metabolic processes and drugs yet not investigated with regard to beige function, such as fasting and exercise mimetics, metabolic drugs and proteins, regulatory circuits of feeding and fasting or circadian rhythm—our data presented here clearly point out the vast potential of they may provide.

## Abbreviations

AgRP: Agouti-related protein
AMPK: AMP-activated protein kinase
BMP4: bone morphogenetic protein 4
CIDEA: cell death-inducing DNA fragmentation factor, alpha subunit-like effector A
FGF21: fibroblast growth factor 21
GLP-1: Glucagon-like peptide-1
hADMSCs: Human adipose-derived mesenchymal stem cells
HIF: hypoxia inducible factor
LSC: Laser scanning cytometry
mTOR: mechanistic target of rapamycin
NAMPT: nicotinamide phosphoribosyl transferase
NRF: nuclear respiratory factor
NRG4: neuregulin 4
PGC-1α: peroxisome proliferator activated receptor cofactor-1α
PPARγ: peroxisome proliferator activated receptor γ
PRDM16: PR domain containing 16
RT-qPCR: reverse transcription-coupled polymerase chain reaction
TBX-1: T-box protein 1
TMEM26: transmembrane protein 26
UCP1: uncoupling protein-1

## References

[1] Finkel T. The metabolic regulation of aging. Nat Med. 2015;21(12):1416–23. 10.1038/nm.3998 26646498

[2] Houtkooper RH, Pirinen E, Auwerx J. Sirtuins as regulators of metabolism and healthspan. Nat Rev Mol Cell Biol. 2012;13(4):225–38. 10.1038/nrm3293 22395773 PMC4872805

[3] Cantó C, Sauve A, Bai P. Crosstalk between poly(ADP-ribose) polymerase and sirtuin enzymes. Molecular Aspects of Medicine. 2013;34(6):1168–201. 10.1016/j.mam.2013.01.004 23357756 PMC3676863

[4] Verdin E, Hirschey MD, Finley LW, Haigis MC. Sirtuin regulation of mitochondria: energy production, apoptosis, and signaling. Trends Biochem Sci. 2010;35(12):669–75. 10.1016/j.tibs.2010.07.003 20863707 PMC2992946

[5] Feige JN, Auwerx J. Transcriptional coregulators in the control of energy homeostasis. Trends Cell Biol. 2007;17(6):292–301. 17475497 10.1016/j.tcb.2007.04.001

[6] Auwerx J. Improving metabolism by increasing energy expenditure. NatMed. 2006;12(1):44–5. 10.1038/nm0106-44 16397563

[7] Wu J, Bostrom P, Sparks LM, Ye L, Choi JH, Giang AH, et al Beige adipocytes are a distinct type of thermogenic fat cell in mouse and human. Cell. 2012;150(2):366–76. 10.1016/j.cell.2012.05.016 22796012 PMC3402601

[8] Petrovic N, Walden TB, Shabalina IG, Timmons JA, Cannon B, Nedergaard J. Chronic peroxisome proliferator-activated receptor gamma (PPARgamma) activation of epididymally derived white adipocyte cultures reveals a population of thermogenically competent, UCP1-containing adipocytes molecularly distinct from classic brown adipocytes. J Biol Chem. 2010;285(10):7153–64. 10.1074/jbc.M109.053942 20028987 PMC2844165

[9] Kazak L, Chouchani ET, Jedrychowski MP, Erickson BK, Shinoda K, Cohen P, et al A Creatine-Driven Substrate Cycle Enhances Energy Expenditure and Thermogenesis in Beige Fat. Cell. 2015;163(3):643–55. 10.1016/j.cell.2015.09.035 26496606 PMC4656041

[10] Kajimura S, Spiegelman BM, Seale P. Brown and Beige Fat: Physiological Roles beyond Heat Generation. Cell Metab. 2015;22(4):546–59. 10.1016/j.cmet.2015.09.007 26445512 PMC4613812

[11] Rosen ED, Spiegelman BM. What we talk about when we talk about fat. Cell. 2014;156(1–2):20–44. 10.1016/j.cell.2013.12.012 24439368 PMC3934003

[12] Pyrzak B, Demkow U, Kucharska AM. Brown Adipose Tissue and Browning Agents: Irisin and FGF21 in the Development of Obesity in Children and Adolescents. Adv Exp Med Biol. 2015;866:25–34. 10.1007/5584_2015_149 26022904

[13] Tharp KM, Jha AK, Kraiczy J, Yesian A, Karateev G, Sinisi R, et al Matrix assisted transplantation of functional beige adipose tissue. Diabetes. 2015. 10.2337/db15-0728 PMC4613967 26293504

[14] Kristof E, Doan-Xuan QM, Bai P, Bacso Z, Fesus L. Laser-scanning cytometry can quantify human adipocyte browning and proves effectiveness of irisin. Sci Rep. 2015;5:12540 10.1038/srep12540 26212086 PMC4515591

[15] Fu T, Seok S, Choi S, Huang Z, Suino-Powell K, Xu HE, et al MicroRNA 34a inhibits beige and brown fat formation in obesity in part by suppressing adipocyte fibroblast growth factor 21 signaling and SIRT1 function. Mol Cell Biol. 2014;34(22):4130–42. 10.1128/MCB.00596-14 25182532 PMC4248715

[16] Christian M. Transcriptional fingerprinting of "browning" white fat identifies NRG4 as a novel adipokine. Adipocyte. 2015;4(1):50–4. 10.4161/adip.29853 26167402 PMC4496975

[17] Gustafson B, Hammarstedt A, Hedjazifar S, Hoffmann JM, Svensson PA, Grimsby J, et al BMP4 and BMP Antagonists Regulate Human White and Beige Adipogenesis. Diabetes. 2015;64(5):1670–81. 10.2337/db14-1127 25605802

[18] Lopez M, Dieguez C, Nogueiras R. Hypothalamic GLP-1: the control of BAT thermogenesis and browning of white fat. Adipocyte. 2015;4(2):141–5. 10.4161/21623945.2014.983752 26167417 PMC4497297

[19] Ruan HB, Dietrich MO, Liu ZW, Zimmer MR, Li MD, Singh JP, et al O-GlcNAc transferase enables AgRP neurons to suppress browning of white fat. Cell. 2014;159(2):306–17. 10.1016/j.cell.2014.09.010 25303527 PMC4509746

[20] McGlashon JM, Gorecki MC, Kozlowski AE, Thirnbeck CK, Markan KR, Leslie KL, et al Central serotonergic neurons activate and recruit thermogenic brown and beige fat and regulate glucose and lipid homeostasis. Cell Metab. 2015;21(5):692–705. 10.1016/j.cmet.2015.04.008 25955206 PMC4565052

[21] Qiang L, Wang L, Kon N, Zhao W, Lee S, Zhang Y, et al Brown Remodeling of White Adipose Tissue by SirT1-Dependent Deacetylation of Ppargamma. Cell. 2012;150(3):620–32. 10.1016/j.cell.2012.06.027 22863012 PMC3413172

[22] Rachid TL, Penna-de-Carvalho A, Bringhenti I, Aguila MB, Mandarim-de-Lacerda CA, Souza-Mello V. Fenofibrate (PPARalpha agonist) induces beige cell formation in subcutaneous white adipose tissue from diet-induced male obese mice. Mol Cell Endocrinol. 2015;402:86–94. 10.1016/j.mce.2014.12.027 25576856

[23] Lee MW, Odegaard JI, Mukundan L, Qiu Y, Molofsky AB, Nussbaum JC, et al Activated type 2 innate lymphoid cells regulate beige fat biogenesis. Cell. 2015;160(1–2):74–87. 10.1016/j.cell.2014.12.011 25543153 PMC4297518

[24] Xu S, Chen P, Sun L. Regulatory networks of non-coding RNAs in brown/beige adipogenesis. Biosci Rep. 2015. 10.1042/BSR20150155 PMC4626868 26283634

[25] Canto C, Gerhart-Hines Z, Feige JN, Lagouge M, Noriega L, Milne JC, et al AMPK regulates energy expenditure by modulating NAD+ metabolism and SIRT1 activity. Nature. 2009;458(7241):1056–60. 10.1038/nature07813 19262508 PMC3616311

[26] Vila-Bedmar R, Lorenzo M, Fernandez-Veledo S. Adenosine 5'-monophosphate-activated protein kinase-mammalian target of rapamycin cross talk regulates brown adipocyte differentiation. Endocrinology. 2010;151(3):980–92. 10.1210/en.2009-0810 20133456

[27] Fischer-Posovszky P, Newell FS, Wabitsch M, Tornqvist HE. Human SGBS cells—a unique tool for studies of human fat cell biology. Obesity facts. 2008;1(4):184–9. 10.1159/000145784 20054179 PMC6452113

[28] Elabd C, Chiellini C, Carmona M, Galitzky J, Cochet O, Petersen R, et al Human multipotent adipose-derived stem cells differentiate into functional brown adipocytes. Stem Cells. 2009;27(11):2753–60. 10.1002/stem.200 19697348

[29] Sullivan JE, Brocklehurst KJ, Marley AE, Carey F, Carling D, Beri RK. Inhibition of lipolysis and lipogenesis in isolated rat adipocytes with AICAR, a cell-permeable activator of AMP-activated protein kinase. FEBS Lett. 1994;353(1):33–6. 7926017 10.1016/0014-5793(94)01006-4

[30] Doan-Xuan QM, Sarvari AK, Fischer-Posovszky P, Wabitsch M, Balajthy Z, Fesus L, et al High content analysis of differentiation and cell death in human adipocytes. Cytometry A. 2013;83(10):933–43. 10.1002/cyto.a.22333 23846866

[31] Schrepfer E, Scorrano L. Mitofusins, from Mitochondria to Metabolism. Mol Cell. 2016;61(5):683–94. 10.1016/j.molcel.2016.02.022 26942673

[32] Wai T, Langer T. Mitochondrial Dynamics and Metabolic Regulation. Trends Endocrinol Metab. 2016;27(2):105–17. 10.1016/j.tem.2015.12.001 Epub 6 Jan 2. 26754340

[33] Bai P, Canto C, Oudart H, Brunyanszki A, Cen Y, Thomas C, et al PARP-1 Inhibition Increases Mitochondrial Metabolism through SIRT1 Activation. Cell Metab. 2011;13(4):461–8. 10.1016/j.cmet.2011.03.004 21459330 PMC3086520

[34] Fodor T, Szántó M, Abdul-Rahman O, Nagy L, Dér Á, Kiss B, et al Combined treatment of MCF-7 cells with AICAR and methotrexate, arrests cell cycle and reverses Warburg metabolism through AMP-activated protein kinase (AMPK) and FOXO1. 2016:resubmitted to PLOS One. 10.1371/journal.pone.0150232 PMC4769015 26919657

[35] Nagy L, Docsa T, Szántó M, Brunyánszki A, Hegedűs C, Márton J, et al Glycogen phosphorylase inhibitor N-(3,5-dimethyl-benzoyl)-N’-(β-D-glucopyranosyl)urea improves glucose tolerance under normoglycemic and diabetic conditions and rearranges hepatic metabolism. PLoS One. 2013;8(7):e0069420. 10.1371/journal.pone.0069420 PMC3723905 23936011

[36] Carlucci A, Lignitto L, Feliciello A. Control of mitochondria dynamics and oxidative metabolism by cAMP, AKAPs and the proteasome. Trends Cell Biol. 2008;18(12):604–13. 10.1016/j.tcb.2008.09.006 18951795

[37] DuBoff B, Feany M, Gotz J. Why size matters—balancing mitochondrial dynamics in Alzheimer's disease. Trends Neurosci. 2013;36(6):325–35. 10.1016/j.tins.2013.03.002 23582339

[38] Zorzano A, Liesa M, Palacin M. Mitochondrial dynamics as a bridge between mitochondrial dysfunction and insulin resistance. Archives of physiology and biochemistry. 2009;115(1):1–12. 10.1080/13813450802676335 19267277

[39] Zorzano A, Liesa M, Palacin M. Role of mitochondrial dynamics proteins in the pathophysiology of obesity and type 2 diabetes. Int J Biochem Cell Biol. 2009;41(10):1846–54. 10.1016/j.biocel.2009.02.004 19703653

[40] de Jong JM, Larsson O, Cannon B, Nedergaard J. A stringent validation of mouse adipose tissue identity markers. Am J Physiol Endocrinol Metab. 2015;308(12):E1085–105. 10.1152/ajpendo.00023.2015 25898951

[41] Hallberg M, Morganstein DL, Kiskinis E, Shah K, Kralli A, Dilworth SM, et al A functional interaction between RIP140 and PGC-1alpha regulates the expression of the lipid droplet protein CIDEA. Mol Cell Biol. 2008;28(22):6785–95. 10.1128/MCB.00504-08 18794372 PMC2573308

[42] Seale P, Bjork B, Yang W, Kajimura S, Chin S, Kuang S, et al PRDM16 controls a brown fat/skeletal muscle switch. Nature. 2008;454(7207):961–7. 10.1038/nature07182 18719582 PMC2583329

[43] Claussnitzer M, Dankel SN, Kim KH, Quon G, Meuleman W, Haugen C, et al FTO Obesity Variant Circuitry and Adipocyte Browning in Humans. N Engl J Med. 2015;373(10):895–907. 10.1056/NEJMoa1502214 26287746 PMC4959911

[44] Imai SI, Guarente L. NAD and sirtuins in aging and disease. Trends Cell Biol. 2014. 10.1016/j.tcb.2014.04.002 PMC4112140 24786309

[45] Bai P, Nagy L, Fodor T, Liaudet L, Pacher P. Poly(ADP-ribose) polymerases as modulators of mitochondrial activity. Trends Endocrinol Metab. 2015;26(2):75–83. 10.1016/j.tem.2014.11.003 25497347

[46] Efeyan A, Comb WC, Sabatini DM. Nutrient-sensing mechanisms and pathways. Nature. 2015;517(7534):302–10. 10.1038/nature14190 25592535 PMC4313349

[47] Valero T. Mitochondrial biogenesis: pharmacological approaches. Curr Pharm Des. 2014;20(35):5507–9. 24606795 10.2174/138161282035140911142118

[48] Canto C, Jiang LQ, Deshmukh AS, Mataki C, Coste A, Lagouge M, et al Interdependence of AMPK and SIRT1 for metabolic adaptation to fasting and exercise in skeletal muscle. Cell Metab 2010;11(3):213–9. 10.1016/j.cmet.2010.02.006 20197054 PMC3616265

[49] Ruderman NB, Xu XJ, Nelson L, Cacicedo JM, Saha AK, Lan F, et al AMPK and SIRT1: a long-standing partnership? Am J Physiol Endocrinol Metab. 2010;298(4):E751–60. 10.1152/ajpendo.00745.2009 20103737 PMC2853213

[50] Um JH, Park SJ, Kang H, Yang S, Foretz M, McBurney MW, et al AMP-activated protein kinase-deficient mice are resistant to the metabolic effects of resveratrol. Diabetes. 2009;59(3):554–63. 10.2337/db09-0482 19934007 PMC2828647

[51] Hou X, Xu S, Maitland-Toolan KA, Sato K, Jiang B, Ido Y, et al SIRT1 regulates hepatocyte lipid metabolism through activating AMP-activated protein kinase. J BiolChem. 2008;283(29):20015–26. 10.1074/jbc.M802187200 PMC2459285 18482975

[52] Fulco M, Cen Y, Zhao P, Hoffman EP, McBurney MW, Sauve AA, et al Glucose restriction inhibits skeletal myoblast differentiation by activating SIRT1 through AMPK-mediated regulation of Nampt. Dev Cell. 2008;14(5):661–73. 10.1016/j.devcel.2008.02.004 18477450 PMC2431467

[53] Fulco M, Sartorelli V. Comparing and contrasting the roles of AMPK and SIRT1 in metabolic tissues. Cell Cycle. 2008;7(23):3669–79. Epub 2008 Dec 9. 19029811 10.4161/cc.7.23.7164 PMC2607479

[54] Vargas D, Rosales W, Lizcano F. Modifications of Human Subcutaneous ADMSC after PPARgamma Activation and Cold Exposition. Stem cells international. 2015;2015:196348 10.1155/2015/196348 26339249 PMC4539182

[55] Shan T, Liang X, Bi P, Kuang S. Myostatin knockout drives browning of white adipose tissue through activating the AMPK-PGC1alpha-Fndc5 pathway in muscle. FASEB J. 2013;27(5):1981–9. 10.1096/fj.12-225755 23362117 PMC3633817

[56] van Dam AD, Kooijman S, Schilperoort M, Rensen PC, Boon MR. Regulation of brown fat by AMP-activated protein kinase. Trends Mol Med. 2015;21(9):571–9. 10.1016/j.molmed.2015.07.003 26271143

[57] Pulinilkunnil T, He H, Kong D, Asakura K, Peroni OD, Lee A, et al Adrenergic regulation of AMP-activated protein kinase in brown adipose tissue in vivo. J Biol Chem. 2011;286(11):8798–809. 10.1074/jbc.M111.218719 21209093 PMC3059037

[58] Ahmadian M, Abbott MJ, Tang T, Hudak CS, Kim Y, Bruss M, et al Desnutrin/ATGL is regulated by AMPK and is required for a brown adipose phenotype. Cell Metab. 2011;13(6):739–48. 10.1016/j.cmet.2011.05.002 21641555 PMC3148136

[59] Wang S, Liang X, Yang Q, Fu X, Rogers CJ, Zhu M, et al Resveratrol induces brown-like adipocyte formation in white fat through activation of AMP-activated protein kinase (AMPK) alpha1. Int J Obes (Lond). 2015;39(6):967–76. 25761413 10.1038/ijo.2015.23 PMC4575949

[60] Zhang H, Guan M, Townsend KL, Huang TL, An D, Yan X, et al MicroRNA-455 regulates brown adipogenesis via a novel HIF1an-AMPK-PGC1alpha signaling network. EMBO Rep. 2015;16(10):1378–93. 10.15252/embr.201540837 26303948 PMC4766451

[61] Xin C, Liu J, Zhang J, Zhu D, Wang H, Xiong L, et al Irisin improves fatty acid oxidation and glucose utilization in type 2 diabetes by regulating the AMPK signaling pathway. Int J Obes (Lond). 2015. 10.1038/ijo.2015.199 26403433

[62] Tchkonia T, Giorgadze N, Pirtskhalava T, Tchoukalova Y, Karagiannides I, Forse RA, et al Fat depot origin affects adipogenesis in primary cultured and cloned human preadipocytes. AmJPhysiol RegulIntegrComp Physiol. 2002;282(5):R1286–R96. 10.1152/ajpregu.00653.2001 11959668

[63] Lefebvre AM, Laville M, Vega N, Riou JP, van GL, Auwerx J, et al Depot-specific differences in adipose tissue gene expression in lean and obese subjects. Diabetes. 1998;47(1):98–103. 9421381 10.2337/diab.47.1.98

[64] Seale P. Transcriptional Regulatory Circuits Controlling Brown Fat Development and Activation. Diabetes. 2015;64(7):2369–75. 10.2337/db15-0203 26050669 PMC4477361

[65] Ye L, Wu J, Cohen P, Kazak L, Khandekar MJ, Jedrychowski MP, et al Fat cells directly sense temperature to activate thermogenesis. Proc Natl Acad Sci U S A. 2013;110(30):12480–5. 10.1073/pnas.1310261110 23818608 PMC3725077

[66] Crane JD, Palanivel R, Mottillo EP, Bujak AL, Wang H, Ford RJ, et al Inhibiting peripheral serotonin synthesis reduces obesity and metabolic dysfunction by promoting brown adipose tissue thermogenesis. Nat Med. 2015;21(2):166–72. 10.1038/nm.3766 25485911 PMC5647161

[67] Oh CM, Namkung J, Go Y, Shong KE, Kim K, Kim H, et al Regulation of systemic energy homeostasis by serotonin in adipose tissues. Nat Commun. 2015;6:6794 10.1038/ncomms7794 25864946 PMC4403443

